# Bridging molecular and cellular neuroscience with proximity labeling technologies

**DOI:** 10.1038/s12276-025-01491-4

**Published:** 2025-07-10

**Authors:** Jun-Gyu Lee, Inyoung Jeong, Kwang-eun Kim

**Affiliations:** 1 Organelle Medicine Research Center, Yonsei University Wonju College of Medicine, Wonju, Republic of Korea; 2 Department of Chemistry, Seoul National University, Seoul, Republic of Korea; 3 Department of Chemistry, Princeton University, Princeton, NJ USA; 4 Department of Convergence Medicine, Yonsei University Wonju College of Medicine, Wonju, Republic of Korea; 5 Department of Global Medical Science, Yonsei University Wonju College of Medicine, Wonju, Republic of Korea

**Keywords:** Protein-protein interaction networks, Molecular neuroscience

## Abstract

Proximity labeling (PL) techniques have advanced neuroscience by revealing the molecular interactions that govern neural circuits. From foundational tools such as BioID and APEX to recent innovations such as TurboID and light-activated systems, PL enables precise mapping of protein–protein interactions within living cells. Recent applications have identified dynamic protein networks in synaptic remodeling, calcium-dependent signaling and disease states, such as neurodegenerative and psychiatric disorders. These studies not only deepen our comprehension of the molecular architecture of the brain but also uncover novel therapeutic targets. By integrating PL with cutting-edge multi-omics strategies and advanced imaging technologies, researchers can decode the intricate interplay between structural and functional neural networks. As PL technologies continue to evolve, they bridge molecular and cellular neuroscience, offering a useful framework for unraveling the complexity of brain networks. Here, in this Review, we underscore the potential of PL in neuroscience, furthering our understanding of the molecular basis of neural connectivity in both health and disease.

## Proximity labeling reveals neural circuit interactions

Understanding how the brain works is a big challenge in neuroscience. This article discusses how researchers are using new methods to better understand these connections and brain functions. The study focuses on a technique called proximity labeling (PL), which helps to identify proteins that interact closely within cells. PL uses special enzymes to tag nearby proteins, making it easier to study their interactions. This method is important because it can capture interactions that other techniques might miss, especially in complex areas like the brain. Researchers have used PL to study different parts of the brain and how they work together. For example, they have identified proteins involved in synapse formation and proteins altered in neurological disorders such as autism. The findings show that PL is a powerful tool for studying the brain’s molecular landscape.

This summary was initially drafted using artificial intelligence, then revised and fact-checked by the author.

## Introduction

Understanding the neural circuits that govern brain function is undoubtedly among the greatest challenges in the realm of neuroscience^1,2^. Connectomes, mapping the structural and functional connections across the brain, are essential for tackling this challenge^3,4^. Recently, the connectome of the computational brain has been shown to offer key insights into brain structure and information processing^5^. These insights have enabled researchers to decode sensorimotor functions and behavioral responses, paving the way for deeper exploration of neural mechanisms. Leveraging detailed connectivity maps and neurotransmitter predictions, researchers can identify circuit components responsible for specific behaviors such as feeding and grooming.

Behavior, development and cognitive functions are underpinned by the molecular diversity and spatial organization of synapses, which have been unveiled through synaptome mapping. For example, high-resolution mapping of the mouse brain synaptome has identified over 1,000 distinct postsynaptic proteins, contributing to the functional heterogeneity of neural circuits^6^. Synaptic diversity is systematically arranged across brain regions, aligning with functional connectome architecture and enabling dynamic processes such as information storage and recall. In addition, synaptome analyses have provided a deeper understanding of how synaptic protein composition is altered in neurological disorders such as autism and schizophrenia, emphasizing the critical role of synaptic integrity in cognitive functions^7,8^. These advances underscore the importance of ‘proteome research’ in elucidating the fundamental principles of neuronal connectivity and disease mechanisms.

To advance this line of inquiry, it is essential to develop methods that can precisely capture the dynamic protein–protein interactions (PPIs) underlying synaptic function. Among mass-spectrometry-based approaches, affinity purification mass spectrometry (AP-MS) is widely regarded as the standard tool for identifying PPIs; however, the milder lysis conditions typically required can impede the capture of membrane proteins. Moreover, weaker or more transient interactions may be lost during the extraction step^9^. Given that synapses are characterized by highly transient and complex protein interactions, these limitations suggest that AP-MS may not provide the high-resolution data needed to fully characterize synaptic PPIs. Consequently, there is a need for improved MS-based methods capable of capturing the rapidly changing and intricate molecular landscape of synapses.

In 2016, Uezu et al.^10^ and Loh et al.^11^ pioneered the proteomic mapping of synapses using proximity labeling (PL) technologies. Since then, PL has emerged as a valuable tool for elucidating molecular network architecture, offering several key advantages over conventional approaches. Notably, PL can be performed in living cells or even live animals, thereby capturing protein interactions under near-physiological conditions while preserving membrane integrity and protein complexes. Importantly, PL enables spatial proteome mapping of otherwise unpurifiable subcellular regions—such as the synaptic cleft^11^—by labeling proteins in situ without the need for physical isolation. This approach circumvents artifacts associated with detergent lysis and enhances the recovery of fragile membrane proteins, including receptors, ion channels and transporters. Leveraging these advantages, PL can be utilized to analyze cell-type-specific spatial proteomes^12^, cell-surface proteomes^13^ and cell–cell interaction networks^14^, shedding light on the intricate molecular and cellular interactions within the brain. It also enables the selective labeling of neighboring cells, facilitating the isolation of target cells for downstream multi-omics analyses, such as single-cell RNA sequencing (scRNA-seq) and proteomics^15,16^. In addition, PL plays a crucial role in uncovering the spatial proteome of the brain and facilitates the integration of multi-omics data. From traditional approaches to cutting-edge methods, PL has notably advanced the study of neural circuits by enabling the mapping of spatial and functional proteomic landscapes^17^.

Here, we review the importance of PL for the network mapping and its applications in neuroscience, emphasizing conceptual advancements. We detail how recent innovations are providing unprecedented insights into dynamic molecular interactions. More technical details can be found elsewhere^18,19^.

## PL techniques

**Fig. 1:**
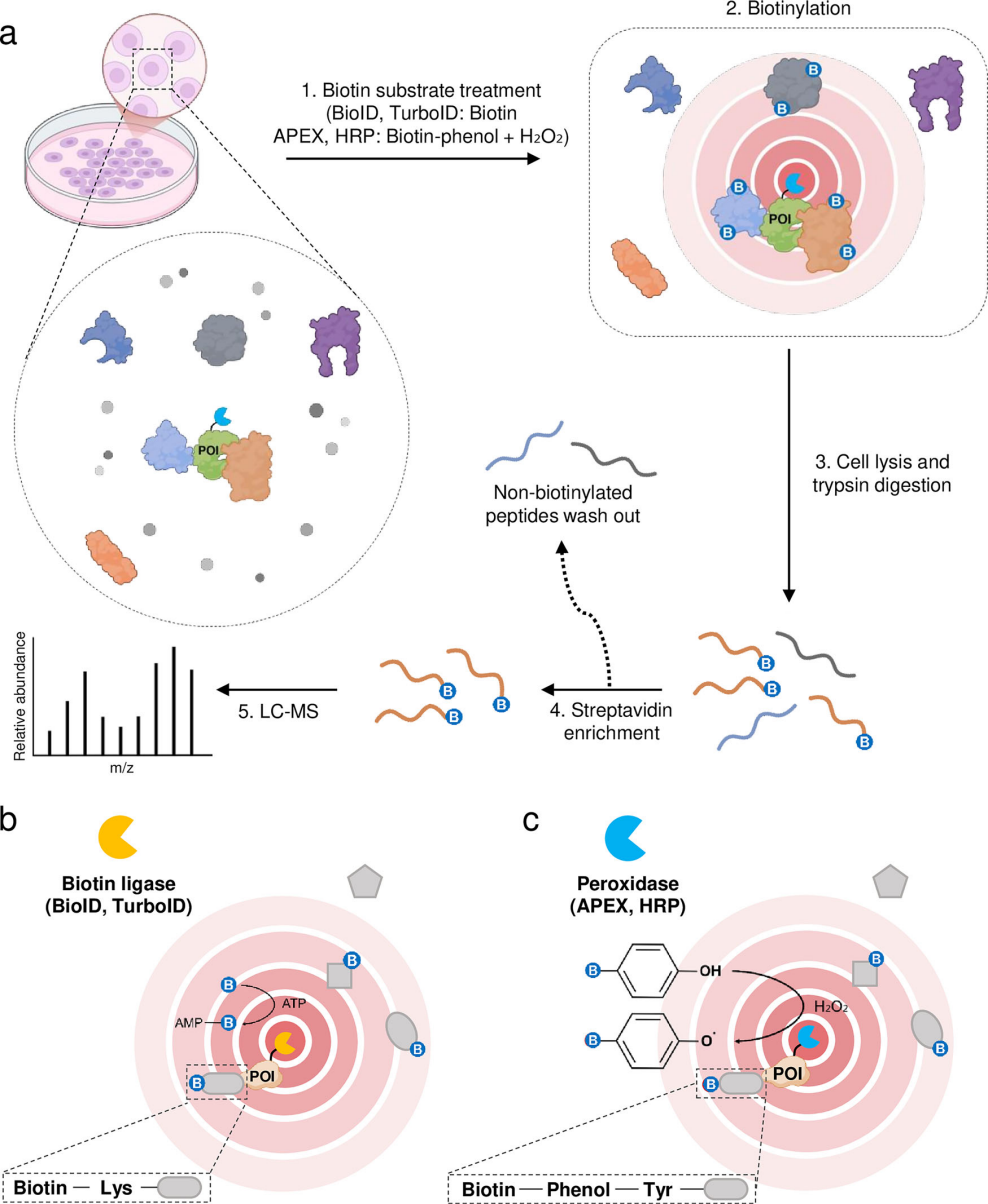
PL workflow and strategies. **a** Cells are genetically engineered to express a fusion protein consisting of a POI and a PL enzyme. The enzyme catalyzes the covalent attachment of a biotin substrate to nearby proteins. After labeling, cells are lysed and enzymatically digested into peptides. Biotinylated peptides are enriched using streptavidin and identified by mass spectrometry analysis. **b** The biotin ligase, activated by ATP, catalyzes the transfer of biotin (B) to nearby proteins, enabling spatially restricted labeling. The POI is fused to the biotin ligase, ensuring selective labeling of proximal proteins. **c** APEX or HRP is fused to a POI to enable localized labeling. In the presence of H_2_O_2_, the peroxidase catalyzes the oxidation of biotin-phenol, generating reactive biotin radicals that covalently label nearby proteins. Created with BioRender.com.

PL has emerged as an effective strategy for investigating PPIs within cells, enabling the detection of interactions that traditional immunoprecipitation-based assays often overlook^20,21^. In this approach, an engineered enzyme fused to a protein of interest (POI) catalyzes the covalent tagging of nearby proteins with a reactive biotin substrate. The biotinylated proteins are then selectively enriched using streptavidin-coated beads and identified via mass spectrometry, enabling a detailed mapping of protein interaction networks within their native cellular environment (Fig. 1a).

The first widely adopted PL system was BioID, which uses a mutated *Escherichia coli* biotin ligase known as BirA. BioID leaks a reactive biotin–AMP intermediate into its surroundings^22,23^ (Fig. 1b). Proteins located within roughly 10 nm of the BirA fusion protein then become biotinylated on lysine residues, enabling their subsequent purification and identification via mass spectrometry. By preserving the native subcellular environment, BioID captures interactions in a physiological context. However, BioID has limitations, including long biotin incubation times (18–24 h) and potential steric hindrance from the relatively large size. To address these challenges, BioID2 was introduced as a smaller, optimized variant of the biotin ligase^24^. This refinement minimizes interference with the target protein’s structure. However, it still generally requires several hours for robust biotin labeling, which can limit its efficiency in certain applications. Particularly in brain or neuron applications, BioID2 has been reported to struggle with accurately detecting neuron-specific interactions^25–27^.

In parallel, APEX, which uses the oxidative activity of a peroxidase, offered a faster alternative. By oxidizing substrates like biotin-phenol (biotinyl tyramide) in the presence of hydrogen peroxide, APEX generates reactive radicals that covalently label nearby proteins within minutes^28^ (Fig. 1c). This kinetic makes it ideal for capturing transient interactions, and its ability to produce electron-dense precipitates facilitates electron microscopy studies^29,30^. However, the use of hydrogen peroxide may induce oxidative stress, thereby causing cytotoxic effects. APEX2, a modified version, improved signal-to-noise ratios by reducing hydrogen peroxide requirements^31^.

Another notable advancement in the PL field is TurboID, created through directed evolution to enhance catalytic activity. TurboID can label proximal proteins within minutes, making it ideal for capturing rapid or dynamic interactions^32^. Its enhanced reactivity, however, can lead to unintended overlabeling and increased background, so it must be carefully calibrated to avoid adversely affecting cell viability^32,33^. Split-TurboID further refines this approach by splitting the TurboID enzyme into two halves, each fused to a different POI^34^. Proximal proteins are labeled only when the two fragments reconstitute TurboID activity through physical interaction, enabling highly specific mapping of PPIs even at organelle contact sites^35^. This approach increases specificity but also adds experimental complexity, requiring precise control over reconstitution kinetics and protein expression.

Overall, PL techniques have enhanced the investigation of protein networks by providing precise and dynamic insights into protein interactions. BioID and BioID2 allow convenient labeling in many cellular compartments but may require lengthy biotin incubations^22,24^. APEX and APEX2 carry out labeling within minutes yet rely on potentially cytotoxic hydrogen peroxide^28,31^. TurboID offers a rapid biotinylation alternative while demanding careful control to avoid background and cell stress^32^. Split-TurboID provides high specificity by reconstituting enzyme activity only upon actual protein–protein contact yet calls for more complex experimental setups and careful optimizations^34^.

TurboID exhibits markedly higher catalytic activity and thus achieves rapid biotinylation within a matter of minutes. However, this enhanced efficiency can cause elevated background labeling. To reduce background labeling, key parameters such as labeling time and biotin concentration must be carefully optimized. In addition, because labeling efficiency and background can vary substantially across different subcellular environments, it is crucial to compare samples within the same compartment using localization-matched constructs. Moreover, incorporating quantitative proteomic approaches—such as tandem mass tag-based labeling—can further refine data interpretation by normalization^32^.

Although negative controls and fold-change calculations offer a practical means of reducing background noise^36^, this statistical approach has inherent limitations in distinguishing true interactors from nonspecifically bound proteins. To overcome this, recent studies have moved toward peptide-level enrichment, which allows direct identification of biotinylation sites and enhances confidence in PPI data^37^. Conventional protein-level enrichment methods often co-purify unlabeled peptides or proteins that are indirectly associated with labeled targets, leading to potential false positives. By contrast, peptide-level enrichment enables direct identification of the biotinylation site, providing strong evidence that the protein was truly labeled in situ. This site-specific information not only increases the confidence in identifying true interactors, but also eliminates the need for negative control-based fold-change calculations. Furthermore, it offers unique advantages such as the ability to infer membrane protein topology and improve the detection of low-abundance proteins that might be masked in protein-level approaches. While peptide-level analysis requires more careful sample preparation and is technically more demanding, its higher specificity and clearer interpretation make it a useful alternative for improving the accuracy of PL-based proteomics.

Another common challenge in biotin-based PL techniques is the presence of endogenously biotinylated proteins, such as carboxylases in mitochondria, which generate strong background signals. In *Caenorhabditis* *elegans*, this issue was addressed by genetically tagging major endogenous biotinylated carboxylases with a His-tag, enabling their selective removal via Ni-based purification^38^. Depleting carboxylases through genetic tagging or antibody-based methods can effectively reduce background noise and enhance the sensitivity of the PL analyses.

It is important to recognize that PL strategies do not definitively demonstrate interactions between bait and neighboring proteins; rather, they provide a qualitative snapshot of spatial proximity^39^. Because biotin ligase-based labeling captures interactions of bait protein within a specific time window (typically 1–16 h), transient or functionally irrelevant associations may be incorporated into the resulting dataset. Therefore, validating PL results using orthogonal approaches—such as co-immunoprecipitation, crosslinking or structural analyses—is essential. Careful experimental design, coupled with rigorous controls, is crucial to ensure that PL-derived data accurately reflect biologically meaningful interaction networks. Biochemists are actively working to overcome the limitations of PL, including the development of novel enzymes^40^.

## Applications in neuroscience

Understanding the brain’s complex molecular architecture requires innovative approaches to study protein interactions and their functional roles. By enabling targeted protein labeling in specific subcellular compartments or cell types, PL has provided insights into synapse composition, traced nascent synapse assembly and mapped proteomic differences across neuronal compartments. Given that synaptic signaling occurs on the millisecond timescale, capturing transient and weak interactions is crucial for elucidating the dynamic molecular events that underlie synaptic function. Therefore, high spatial and temporal resolution are essential. PL techniques overcome the limitations of conventional proteomic methods by allowing in situ labeling of protein networks with spatiotemporal precision, making them particularly suited for studying the rapidly evolving proteomic landscape of synapses^41^. Furthermore, cell-type-specific PL strategies have advanced our understanding of how astrocytes, microglia and neurons interact to maintain brain homeostasis. Below, we provide an overview of the applications of PL in neuroscience, highlighting its potential for advancing our understanding of brain function and disease (Table 1).

**Table 1:** Overview of PL applications in neuroscience.

PL application	Target cell or region	Subcellular localization	Model	Main finding	REF #
PPI	Cortex and hippocampus	Inhibitory postsynaptic density	▪AAV-EF1a- 1) BirA-gephyrin 2) PSD-95-BirA	InSyn1: mediating postsynaptic inhibition	^10^
PPI	Primary neurons	Cytosol, synaptic regions	▪pLV-hSyn-tGFPP2A- POI-Linker-BioID2-FLAG	Mapped PPI networks of 41 ASD risk genes, highlighting mitochondrial and synaptic processes	^25^
PPI	Cortex and hippocampus	Excitatory synapse	▪AAV-EF1a-Wrp-BirA	CARMIL3: regulating synaptic maturation	^42^
PPI	Human neurons	Synaptic vesicles, mitochondria	▪AAVS1-CAG-rtTA3G-TRE3G- 1) APEX-Tau 2) Tau-APEX 3) APEX-α-Tubulin	Revealed activity-dependent Tau–mitochondria interaction changes, linked to neurodegeneration	^45^
Subcellular proteomics	Synapses (excitatory and inhibitory)	Synaptic cleft	▪pLV-HRP- 1) LRRTM1 2) LRRTM2 3) NLGN2 4) Slitrk3	Characterized the distinct proteomes of excitatory and inhibitory synaptic clefts and identified Mdga2 as a synaptic specificity factor	^11^
Subcellular proteomics	mDA neuron VM, MFB, striatum	Cytoplasm	▪DAT-IRES-Cre ▪AAV-CAG-DIO-APEX2-NES	Somatodendritic and axonal proteome Dopaminergic presynapse proteome	^43^
Subcellular proteomics Cell-type-specific proteomics	Direct spiny projection neuron Indirect spiny projection neuron	Nucleus, cytoplasm, plasma membrane	▪Drd1-Cre ▪A2a-Cre ▪AAV-Ef1a-DIO-1) H2B-APEX2 2) APEX2-NES 3) LCK-APEX2	Dynamic proteome of SPNs	^12^
Cell-type-specific proteomics	Excitatory glutamatergic neuron Inhibitory GABAergic neuron Corticostriatal projection neuron	Cytoplasm	▪Vglut2-Cre ▪Vgat-Cre ▪Rbp4-Cre ▪DIO-APEX2-NES Tg	Temporal proteome of corticostriatal axon	^48^
Cell-type-specific proteomics	Neuron and astrocyte	Whole cell	▪AAV-Camk2a-TurboID ▪AAV-GFAP-TurboID	Differences in neuron and astrocyte proteome	^49^
Cell-type-specific proteomics	Microglial and neuronal cell lines	Cytosol	▪pLV-EF1-TurboID-NES	Biotinylated cytosolic proteins with minimal impact on baseline cellular function	^51^
Cell-type-specific proteomics	Cortex and hippocampus	Mitochondria, synapse, cytoskeleton	▪LoxP-Stop-LoxP-TurboID-NES Tg ▪AAV-PHP.eB-E2-Cre-2A-GFP	Mitochondrial dysfunction in PV-INs; synaptic protein loss in early AD pathology	^52^
Cell-type-specific proteomics Region-specific proteomics	Neuron and astrocyte Cortex/hippocampus, Striatum/thalamus Pons/medulla Cerebellum	Cytoplasm	▪LoxP-Stop-LoxP-TurboID-NES Tg ▪Camk2a-Cre/ERT2 ▪Aldh1l1-Cre/ERT2	Differences in neuron- and astrocyte-derived signaling	^50^
Region-specific proteomics	Mouse brain cells	Multiple compartments	▪AAV-PHP.eB-Lrrc4c-TurboID ▪AAV-PHP.eB-Syngap1-TurboID	Revealed interactions between high-confidence ASD risk genes and novel modifier proteins	^44^
Surface proteomics	Astrocyte-secreted synaptic regions	Inhibitory synapse ECM	▪AAV-PHP.eB-GfaABC1D-IgK 1) TurboID-NCAN IL 2) TurboID-NCAN ELS	Identified neurocan C-terminal fragment controlling somatostatin^+^ inhibitory synaptogenesis	^53^
Surface proteomics	Purkinje cell	Plasma membrane	▪Pcp2-Cre LoxP-Stop-LoxP-HRP-PDGFRb	Armh4: regulating dendrite morphogenesis	^54^
Surface proteomics Cell–cell interaction	Astrocyte–neuron	Plasma membrane	▪AAV-hSyn-N-TurboID-GPI ▪AAV-GfaABC1D-C-TurboID-GPI	NRCAM: controlling inhibitory synaptic organization	^56^
Cell–cell interaction	Striatal astrocytes and neurons	Cytosol, plasma membrane endfeet	▪AAV2-GfaABC1D- 1) AQP4-BioID2 2) EZR-BioID2 3) GLT1-BioID2 4) KIR4.1-BioID2 5) CX43-BioID2 ▪AAV-GfaABC1D-BioID2-SAPAP3 ▪AAV-hSyn1-BioID2-SAPAP3	Differentiated astrocytic and neuronal proteomes; identified SAPAP3 as a key modulator in obsessive–compulsive disorder-related phenotypes	^46^

AD, Alzheimer’s disease; PV-INs, Parvalbumin-expressing interneurons.

### Spatial neuroproteomics by PL

Neurons exhibit highly polarized structures, with distinct protein–protein networks in compartments such as dendrites and axons that are not easily captured by traditional methods. Inhibitory synapses, for example, are particularly important for regulating neuronal excitability through hyperpolarizing postsynaptic cells, yet inhibitory postsynaptic density (iPSD) proteins remain relatively uncharacterized. Seeking to address this knowledge gap, Uezu et al.^10^ used an in vivo BioID (iBioID) approach by expressing a gephyrin–BirA fusion via adeno-associated virus (AAV) in mouse brains, identifying 181 biotinylated proteins at the iPSD. Among these, the newly discovered InSyn1 was found to interact with the dystrophin complex and modulate GABAergic inhibition, suggesting a pivotal link between iPSD protein networks and inhibitory synaptic functionality.

On the excitatory side, Spence et al.^42^ overcame the challenge of studying transient nascent synapses during early postnatal development by fusing Wrp to BirA, which specifically labeled proteins in dendritic filopodia. Their screen identified 60 proteins, including CARMIL3, an actin-regulating protein whose depletion impaired AMPA receptor (AMPAR) recruitment and synaptic transmission. These findings collectively highlight how cytoskeletal remodeling can drive synapse formation and maturation in excitatory circuits.

Moving beyond synapse assembly, PL has proven critical for dissecting proteome differences in subcellular compartments of specialized neuronal populations. Hobson et al.^43^ utilized an APEX2-mediated PL strategy to map subcellular proteomes of midbrain dopaminergic (mDA) neurons in mouse brain slices. They revealed that axons in the striatum harbored the majority of mDA-neuron-specific proteins involved in metabolism, neurotransmission and proteostasis. These findings underscore the morphologically elaborate nature of mDA neurons, offering fresh insights into axonal specialization and potential vulnerabilities in neurodegenerative disorders such as Parkinson’s disease. By illuminating how distinct compartments within the same neuron can display different protein complements, PL approaches refine our understanding of neuronal complexity at an unprecedented resolution.

Building on this progress, PL techniques are increasingly being applied to uncover the molecular underpinnings of diverse neurological disorders. Murtaza et al.^25^ harnessed BioID2 to identify PPI networks of 41 autism spectrum disorder (ASD) risk genes, unveiling convergent pathways tied to mitochondrial function and synaptic transmission. Along the same lines, Gao et al.^44^ used TurboID-driven CRISPR PL to map the spatial proteomes of 14 high-confidence ASD genes in brain tissue, revealing disruptions in glutamatergic signaling and cytoskeletal organization. These large-scale analyses advance our grasp of how overlapping molecular pathways contribute to ASD pathophysiology.

Tracy et al.^45^ integrated APEX with AP-MS in human neurons to examine Tau interactomes. Their findings show that Tau interacts with synaptic vesicle and mitochondrial proteins in an activity-dependent manner and that frontotemporal-dementia-linked Tau mutations disrupt these interactions. Meanwhile, Soto et al.^46^ leveraged BioID2 to chart astrocyte and neuron subproteomes in the striatum, focusing on the actin regulator SAPAP3. They revealed that SAPAP3 in astrocytes and neurons plays distinct roles in anxiety-like and repetitive behaviors. This suggests that obsessive–compulsive disorder phenotypes may result from dysregulation across multiple cell types rather than neurons alone.

Taken together, these studies illustrate the versatility and depth of insights offered by PL for spatial neuroproteomics. To enable spatially resolved neuroproteomics, the choice of PL enzymes is critical. TurboID and miniTurbo offer rapid biotinylation under physiological conditions with low cytotoxicity, making them well suited for in vivo applications. Unlike AP-MS, which often misses transient or weak interactions due to harsh lysis conditions, TurboID-based PL can capture these interactions in situ with higher spatial and temporal fidelity^47^. By contrast, APEX2 offers superior spatial resolution at the subcellular level; however, its reliance on hydrogen peroxide limits its applicability in live mice owing to potential toxicity. Therefore, TurboID-based AAV systems are preferred for spatial neuroproteomics in vivo, whereas APEX2 is advantageous for high-resolution mapping in fixed or ex vivo samples. PL approaches have unveiled molecular mechanisms underlying synaptic development, compartmental specialization and pathology in various brain disorders. They achieve this by capturing interactions in their native cellular context. As a result, these research papers not only resolve longstanding questions about subcellular protein organization but also pave the way for potential therapeutic strategies that selectively target disease-relevant interactions.

### Cell-type-specific spatial proteomics by PL

**Fig. 2:**
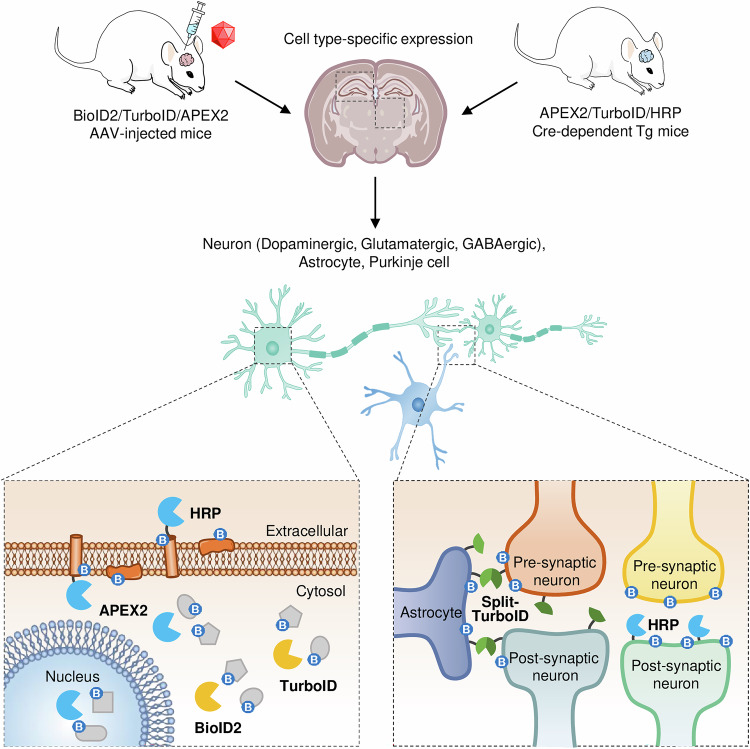
Application of PL in neuroscience. This schematic illustrates how PL enzymes are used to study spatially resolved proteomic interactions in the nervous system. The top panel depicts two major strategies for PL enzyme delivery: a Cre-independent method using AAVs driven by cell-type-specific promoters, and a Cre-dependent method using floxed PL constructs in Cre-transgenic animals. These approaches allow selective expression of enzymes such as APEX, BioID2, TurboID or HRP in specific neuronal or glial populations. Once expressed, PL enzymes can be targeted to distinct subcellular compartments—for example, cytosolic TurboID enables broad labeling within the cytoplasm of defined cells. The bottom panel illustrates the use of PL for probing intercellular proteomic environments, including synaptic interfaces. Split-TurboID, in particular, enables selective labeling at cell–cell contact sites by reconstituting enzyme activity when complementary fragments are expressed in interacting cell types, such as neurons and astrocytes, allowing precise mapping at the tripartite synapse.

Neurons and glial cells each possess uniquely specialized proteomes, driving a wide range of physiological processes. To delineate these cell-type-specific features more precisely, recent studies have used PL techniques such as APEX2 and TurboID. By enabling targeted labeling of distinct subcellular compartments, these methods reveal how neuronal and glial proteomes are organized and how they respond to various stimuli (Fig. 2).

Dumrongprechachan et al.^12^ provided an early demonstration of how APEX2 can be directed to specific subcellular regions to profile the proteomes of spiny projection neurons (SPNs) within the corticostriatal circuit. By expressing APEX2 constructs in the nucleus, cytoplasm or membrane, they captured distinct subcellular proteomes in both direct and indirect SPNs, while also tracking dynamic changes induced by selective activation of striatal neurons using a designer Gq-coupled human M3 receptor (hM3Dq). This DREADD (Designer Receptors Exclusively Activated by Designer Drugs) increases neuronal excitability via Gq-protein signaling in response to the synthetic ligand clozapine-*N*-oxide. Among these changes, proline-directed kinases stood out for their roles in axonal growth and broader neurodevelopmental processes.

Furthermore, in a separate study, Dumrongprechachan et al.^48^ introduced a Cre-dependent APEX2 reporter mouse, allowing cell-type-specific APEX2 expression in either glutamatergic or GABAergic neurons. Notably, this system enabled protein labeling in ex vivo brain slices within just 1 h, a notable reduction compared with the extended durations needed for traditional BioID experiments. Despite the advantages of rapid labeling and high spatial resolution, the reliance on hydrogen peroxide and the limited penetration of biotin-phenol remain constraints for in vivo APEX2 applications.

To address these limitations, TurboID has emerged as a complementary PL platform. Sun et al.^49^ used AAV-mediated TurboID delivery to profile neuron- and astrocyte-specific proteomes, capturing approximately 10,000 unique proteins. Their data point to a richer understanding of cell-type-specific signaling pathways and molecular functions in situ. Rayaprolu et al.^50^ developed a TurboID-based mouse model for profiling neurons and astrocytes with high specificity. They identified more than 2,000 proteins for each cell type, revealing distinct proteomic signatures across different brain regions. Sunna et al.^51^ extended TurboID’s utility to include microglia, thereby distinguishing neuronal and microglial proteomic profiles and uncovering how these two cell types respond differently to inflammatory stimuli.

Likewise, Kumar et al.^52^ used a cell-type-specific in vivo biotinylation approach to analyze the native-state proteome of parvalbumin interneurons, identifying over 600 enriched proteins. Their study highlighted unique molecular signatures of these neurons and revealed vulnerabilities in mitochondrial function and synaptic integrity during early Alzheimer’s pathology. Thanks to TurboID’s rapid labeling and adaptability, these studies highlight unique protein networks and the dynamic interplay among diverse brain cell types.

Collectively, these investigations uncover the considerable impact of PL approaches in unraveling cell-type-specific proteomic landscapes. Through APEX2’s precise subcellular targeting and TurboID’s rapid labeling, researchers can chart the molecular signatures of neurons, astrocytes and microglia in vivo. These insights deepen our understanding of cellular function at the molecular level and uncover previously unrecognized vulnerabilities that may contribute to neurological diseases.

### Cell-surface proteome mapping by PL

Cell-surface proteins are critical for many brain processes, including excitatory and inhibitory synaptic signaling and neuron–glia interactions. However, conventional membrane extraction methods tend to either introduce intracellular contaminants or are unable to capture transient interactions. PL has developed into an invaluable strategy to define the composition of cell-surface proteomes in more physiological conditions. PL can be carried out using various enzymes—such as horseradish peroxidase (HRP), TurboID and split-HRP—depending on the experimental design and goals.

Loh et al.^11^ demonstrated how an HRP-based approach could distinguish the cell-surface proteomes of excitatory and inhibitory synapses by tagging HRP to specific synaptic proteins in cultured cortical neurons. Their findings underscored the utility of PL in identifying synaptic cleft proteins that are uniquely associated with excitatory versus inhibitory signaling. Irala et al.^53^ used TurboID to investigate how astrocyte-secreted extracellular matrix proteins regulate inhibitory synapse formation. By fusing TurboID to neurocan, they identified a C-terminal fragment that modulates somatostatin-positive inhibitory synapses, uncovering key pathways through which glial cells influence neuronal connectivity at the cell surface.

Focusing on developmental dynamics, Li et al.^13^ applied an HRP-based PL strategy in *Drosophila* projection neurons to track how the cell-surface proteome changes across developmental stages. One notable finding was a new role for LRP1 in guiding dendrite targeting, demonstrating how PL can pinpoint molecules crucial for neural circuit wiring. In the mammalian system, Shuster et al.^54^ introduced iPEEL, another HRP-dependent method, to profile the cell-surface proteome of Purkinje cells during cerebellar development. Their work identified Armh4, a transmembrane protein that is essential for dendritic morphogenesis, thus illustrating the power of PL to uncover previously uncharacterized regulators of neuronal growth.

Building upon these advances, super-resolution PL (SR-PL) is emerging as a powerful tool for refining cell-surface proteome studies. Unlike conventional methods that capture both labeled and unlabeled peptides, SR-PL isolates only biotinylated peptides, providing precise site-specific information^37,55^. Conventional statistical approaches often result in false positives, whereas SR-PL directly identifies them. This peptide-level resolution provides critical insights into spatial orientation or topology, such as distinguishing whether modified residues face the cytosol or surface. Applying SR-PL to neuronal systems could further elucidate the spatial arrangement of membrane-associated signaling complexes and the topological organization of neuron–glia interfaces. This promises a new level of precision in understanding neural connectivity.

Collectively, these studies emphasize that PL can reveal cell-surface molecules and interactions that drive synapse organization, neuronal differentiation and neuron–glia communication. By illuminating how cell-surface protein networks contribute to fundamental processes, this line of research also offers new insights into the pathophysiology of neurological disorders.

### PL application for cell–cell interaction network studies

Cell–cell interactions in the central nervous system are of great importance owing to the highly diverse and complex network of cells present. Nevertheless, elucidating intercellular communication has remained challenging because of the scarcity of specialized tools and the low spatial or temporal resolution of conventional approaches. Recent developments of PL have enabled the study of cell–cell communications in the brain and immune system with unprecedented resolution.

Takano et al.^56^ utilized the split-TurboID technique and they identified NRCAM at astrocyte–neuron junctions and confirmed its transcellular interaction with neuronal gephyrin at inhibitory synapses. They suggested that astrocytic NRCAM plays a critical role in inhibitory synapse formation by interacting with gephyrin, a postsynaptic scaffold protein essential for stabilizing GABAergic synapses. This study highlights the regulatory influence of astrocytes on synaptic specialization and stabilization, emphasizing the potential of PL tools in revealing molecular mechanisms at tripartite synapses. Furthermore, this approach can be connected to the historical exploration of ATP as a neurotransmitter in neuron–glia communication and synaptic regulation^57^. This suggests that ATP itself could serve as a valuable PL target, enabling high-resolution mapping of purinergic signaling at cell–cell junctions.

**Fig. 3:**
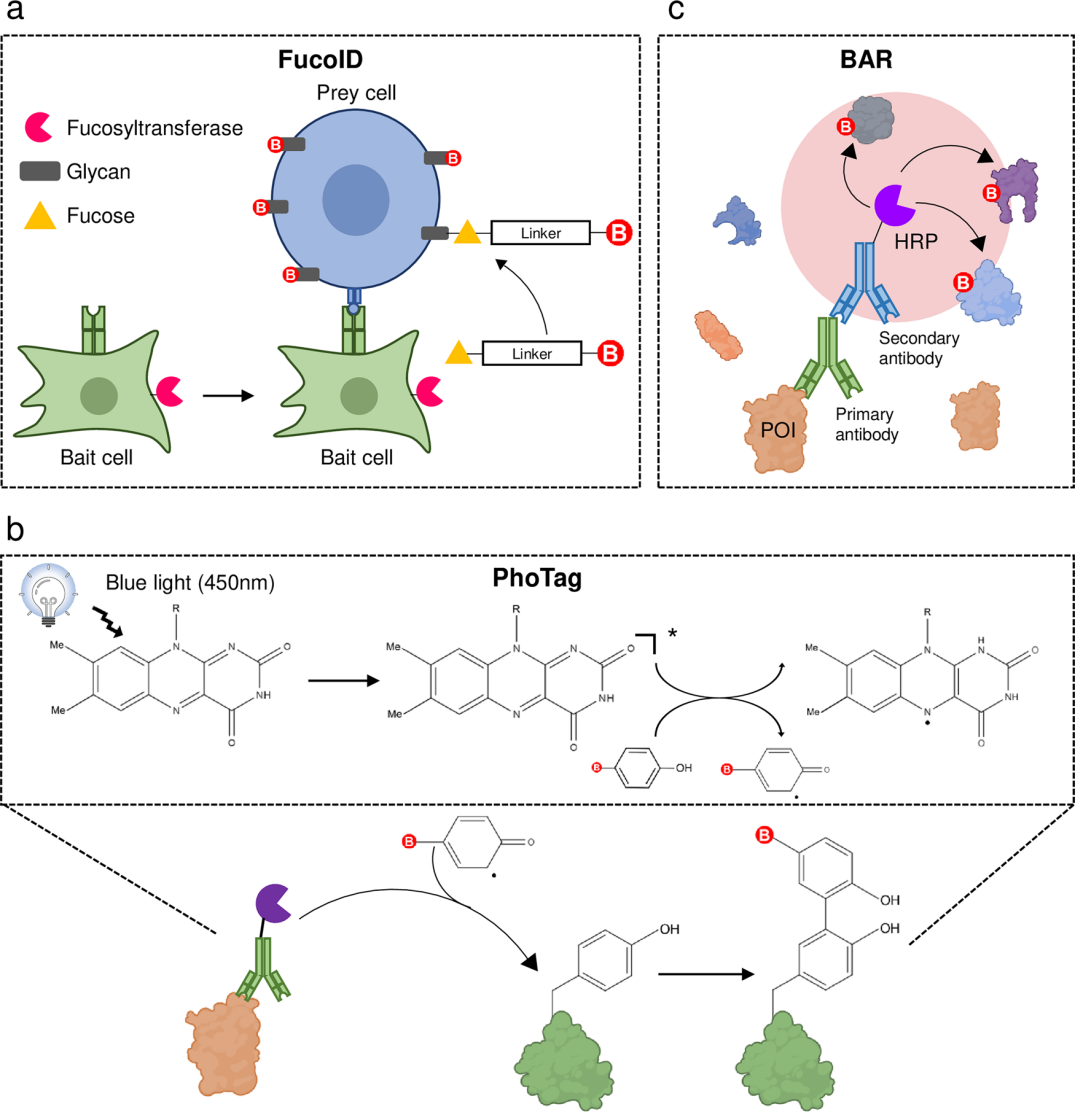
Emerging PL technologies. **a** FucoID is a glycosyltransferase-based PL method used to detect cell–cell interactions. A recombinant FT is conjugated to a bait cell via a targeting antibody. When the bait cell engages a prey cell through specific membrane protein interactions—such as MHC–TCR binding—the resulting cell–cell contact allows FT to transfer a biotinylated fucose analog (GDP-Fuc-Biotin) to glycoproteins on the adjacent prey cell surface. This enables selective biotinylation of interacting cells in a contact-dependent manner, facilitating their identification and enrichment. **b** PhoTag is a photocatalytic cell tagging strategy that utilizes a flavin-based photocatalyst conjugated to an antibody targeting a membrane POI. Upon blue-light irradiation (*λ* = 450 nm), the photocatalyst activates biotin-tyramide to generate phenoxy radicals, which selectively label solvent-exposed tyrosine residues on nearby proteins at the cell surface. **c** BAR is a PL method in which HRP is conjugated to a secondary antibody that binds to a primary antibody recognizing the POI. In the presence of hydrogen peroxide (H_2_O_2_) and biotin-tyramide, HRP catalyzes the formation of highly reactive biotin-phenoxyl radicals that covalently label tyrosine residues on nearby proteins. This enables spatially restricted, antibody-guided proteomic mapping of endogenous targets without genetic manipulation.

Moreover, other PL enzymes have been developed to study cell–cell interactions in other biological systems^58^. Pasqual et al.^59^ introduced an in vivo cell–cell interaction labeling technique using sortase A, which transfers biotin-LPXTG to the proximal N-terminal oligoglycine. This method successfully monitored interactions between CD4^+^ T cells and dendritic cells. Similarly, FucoID, developed by Liu et al.^60^, utilized fucosyl transferase to label tumor-specific antigen-reactive T cells by transferring fucose-biotin to proximal glycan (Fig. 3a). Fucosyltransferase (FT)-conjugated dendritic cells co-cultured with tumor cell suspensions specifically labeled interacting CD4^+^ and CD8^+^ T cells, which were subsequently analyzed via flow cytometry and RNA sequencing. These examples illustrate the effectiveness of PL approaches in immune systems and their adaptability for broader applications.

These advancements collectively showcase the versatility and transformative potential of PL tools in mapping cell–cell interaction networks across diverse biological contexts. Although demonstrated primarily in immune and tumor models, newly developed PL tools can also be applied to the central nervous system to elucidate the intercellular brain network.

## Emerging techniques and future directions

Recently, newer approaches—photocatalytic PL and calcium-dependent PL—have offered improved spatial and temporal resolution while reducing background noise. Moreover, the integration of PL with multi-omics or imaging techniques represents a promising direction, enabling a more comprehensive understanding of cellular processes. In addition, the expansion of PL into phosphoproteomics holds potential for uncovering dynamic phosphorylation events and signaling networks. Below, we provide an overview of these emerging techniques and future directions, highlighting their benefits and opportunities in advancing biomedical research (Table 2).

**Table 2:** Emerging PL Technologies.

	Molecule	Type	Labeling time	Advantages	Limitations	Reference
Photocatalytic PL	PhoxID	Affinity-based photoactivation	1 min	Direct in vivo use, receptor targeting	Probe delivery and light penetration limitations	^69^
	PDPL	Photosensitizer-based labeling	~2–20 min	High specificity, spatiotemporal precision	Requires miniSOG fusion, diffusion limited	^64^
	PhoTag	Photocatalytic cell tagging	~2–10 min	Nongenetic and noninvasive labeling with high spatial–temporal control	Requires light exposure for activation	^70^
Calcium-dependent PL	CaST	Biotin ligase	~10–30 min	Rapid and precise Ca^2+^-triggered labeling	Requires exogenous biotin and Ca^2+^ influx	^73^
	Cal-ID	Biotin ligase	~5–15 min	Rapid and precise Ca^2+^-triggered labeling	Requires exogenous biotin and Ca^2+^ influx	^74^
Phosphoproteomics	BioID – CK2 BioID – PKA	Biotin ligase - kinase fusion protein	24 h	Detection of kinase-specific phosphorylation	Long labeling time	^83^
	APEX2 – MAPK1 APEX2 – PKA	Peroxidase - kinase fusion protein	1 min	Rapid detection of phosphorylated substrates with spatial resolution	Requires H_2_O_2_ which can perturb redox-sensitive processes	^81^

### Photocatalytic PL

Photocatalytic PL exploits photosensitizers that generate reactive species (singlet oxygen, carbenes or radicals) upon illumination with light of appropriate wavelengths^61^. The resultant reactive intermediates then react quickly with nearby proteins, enabling highly localized labeling. Because these species have a short diffusion radius (2–4 nm in the μMap^62^), the labeling remains confined to the immediate vicinity of the photosensitizer, reducing off-target effects.

A common strategy involves genetically encoding a photosensitizer, like miniSOG fused directly to the POI^63,64^. When illuminated by blue light, miniSOG converts molecular oxygen into singlet oxygen, selectively labeling proteins in close proximity^64^. Notably, singlet oxygen preferentially reacts with electron-rich residues such as histidine, tyrosine and tryptophan. Because these aromatic residues are often located at functionally critical sites, singlet oxygen-mediated labeling provides enhanced specificity for capturing biologically meaningful protein interactions^65^. This setup offers precise temporal control, making it particularly effective for capturing dynamic protein interactions. However, blue or green light penetrates tissues poorly in vivo owing to scattering effects and overlap with endogenous chromophore absorption^66^. To overcome these obstacles, red-shifted photosensitizers such as μMap-Red^67^ that respond to near-infrared wavelengths are being developed, mitigating cellular damage and improving penetration depth^68^.

Small-molecule photosensitizers provide another route for photocatalytic labeling. For example, PhoxID^69^ uses a ligand–photosensitizer conjugate that targets endogenous neurotransmitter receptors such as AMPARs. Upon green-light exposure (approximately 520 nm), the photosensitizer produces singlet oxygen to covalently modify nearby proteins. This rapid labeling process can capture receptor-proximal proteomes in living tissue within minutes, revealing how receptor complexes change under different conditions. Despite these advantages, delivery of the photosensitizer and the need for optical fiber insertion in deep tissues remain challenges.

Another emerging photocatalytic PL approach is PhoTag^70^, which utilizes visible light activation of flavin-based cofactors conjugated to single-domain antibodies (Fig. 3b). Upon illumination with blue light (450–470 nm), the flavin photocatalyst generates phenoxy radicals that covalently label proximal tyrosine residues, allowing precise mapping of cell–cell interactions^70^. A key advantage of PhoTag is its spatiotemporal control, enabling targeted labeling with minimal cellular disruption.

Combining photocatalytic PL with optogenetics could enable precise mapping of dynamic protein interactions in neuroscience, improving the resolution of interaction networks in living systems. Unlike traditional PL methods with broader labeling radii, photocatalytic PL generates short-lived radicals that label proteins within just a few nanometers, reducing background and enhancing localization accuracy. In addition, light-based activation enables fine temporal control, as miniSOG, PhoxID and PhoTag require relatively short irradiation times—typically within 20, 10 and 15 min, respectively. Because labeling is light-triggered, these systems are switchable and reversible, allowing researchers to initiate and terminate labeling. By tuning the intensity and duration of light exposure, these systems allow precise capture of transient and activity-dependent protein interactions with minimal perturbation, making them especially suitable for studying dynamic processes in the nervous system. These features make photocatalytic PL well suited for investigating complex and dynamic protein networks in the nervous system.

### Calcium-dependent PL

Calcium surges play critical roles in synaptic plasticity, neurotransmission and memory formation^71,72^. Standard PL techniques are poorly suited for monitoring rapid fluctuations; therefore, calcium-dependent PL is designed to capture fast, transient signaling events. By engineering PL enzymes that activate in response to local calcium level increases, researchers can capture a biochemical snapshot at the precise moment of the calcium spike.

One innovative approach, called Ca^2+^-activated Split-TurboID (CaST)^73^, leverages a split-TurboID system that reassembles into an active enzyme only in the presence of elevated calcium levels. This system comprises two inactive fragments linked to calmodulin or its target peptide M13, which reassemble upon calcium binding to biotinylate nearby proteins. By allowing rapid, transient and calcium-dependent labeling of cellular events within a precise temporal window, CaST captures molecular interactions with high temporal specificity. Unlike traditional calcium sensors needing optical input, CaST provides a stable biochemical record of calcium signals with systemic biotin delivery in freely moving organisms.

Another strategy, referred to as Cal-ID^74^, fuses a calcium indicator with TurboID in circular form. In this system, when calcium indicator detects elevated calcium, the Cal-ID labels proximal proteins with biotin. Cal-ID offers a molecular record of calcium signaling events, allowing spatial mapping of calcium activity over extended periods. Furthermore, Cal-ID enables the identification of key calcium-regulated proteins with minimal perturbation to cellular function. Cal-ID successfully captured calcium-induced biotinylation upon neuronal activation, particularly highlighting plasma membrane calcium ATPases as major targets in primary cortical neurons. Moreover, in vivo experiments demonstrated that kainic-acid-induced seizures triggered calcium-dependent biotinylation, validating its ability to record neuronal activity in complex brain tissues.

Given the pivotal role of calcium in neuronal function, calcium-dependent PL systems like CaST and Cal-ID serve as a useful approach for neuroscience research. These methods enable precise spatiotemporal mapping of calcium microdomains, allowing researchers to investigate dynamic calcium signaling at subcellular scales. Moreover, by overcoming the limitations of traditional calcium indicators, these methods can reveal key molecular regulators involved in calcium-mediated neural processes and brain disorders in mouse models.

### Biotinylation by antibody recognition

Biotinylation by antibody recognition (BAR) provides an effective alternative to genetically encoded PL systems, particularly in experimental contexts where genetic manipulation is impractical—such as in fixed tissues and postmortem human brain samples (Fig. 3c). Unlike methods such as TurboID or APEX that require the expression of fusion proteins, BAR relies on antibodies to guide HRP to the POI, where it catalyzes the labeling of biotin onto proximal proteins in the presence of hydrogen peroxide^75^. This antibody-directed enzymatic labeling enables proteomic profiling of endogenous protein environments without requiring transgene expression.

The utility of BAR has been demonstrated in neuroscience. For instance, Ogawa et al.^76^ applied BAR to map the extracellular proximity proteome of the axon initial segment in cerebellar and cortical neurons, uncovering Contactin-1 as a key regulator of inhibitory axo-axonic synapses. Likewise, Killinger et al.^77^ utilized BAR to dissect the molecular architecture in formalin-fixed human brain tissue, identifying protein networks associated with pathological alpha-synuclein relevant to Parkinson’s disease and dementia with Lewy bodies. These studies highlight BAR’s capacity to uncover biologically meaningful interactions in intact human tissues that are otherwise inaccessible to conventional PL approaches.

Nonetheless, BAR presents several limitations. Its dependence on high-quality, well-validated antibodies restricts its application to well-characterized proteins. Furthermore, the labeling radius (200–300 nm) is broader than that of genetically encoded enzymes like TurboID or APEX2, which can reduce spatial specificity. The requirement for hydrogen peroxide, although suitable for fixed samples, also limits the method’s compatibility with live-cell applications owing to potential cytotoxicity. To address these limitations, microscopy-guided techniques have been developed, and commercial implementations of this approach are available^78^. By leveraging high-resolution imaging and photoactivatable biotin probes, Microscoop Mint (Syncell) enables submicron-scale, light-directed labeling, thereby enhancing spatial specificity and reducing off-target background—particularly in complex or fixed tissue samples.

### Phosphoproteomics

Decoding intricate signaling pathways largely depends on studying posttranslational modifications, especially phosphorylation. Phosphoproteomics has become an important method for capturing these signaling events, as phosphorylation regulates major processes including signal transduction, cell proliferation and differentiation. For example, proteogenomic analysis of glioblastoma progression demonstrated that recurrent tumors adopt a neuronal phenotype driven by phosphorylation and pathway activation, contributing to therapeutic resistance^79^. Moreover, integrating global protein expression and glycosylation data with phosphorylation offers a comprehensive view of disease-associated protein networks, emphasizing the role of phosphoproteomics in understanding disease evolution^80^.

Traditional phosphoproteomics methods face challenges in achieving spatial and temporal resolution, but recent advances have adapted PL for phosphoproteomics^47^. The Phospho-APEX (pAPEX)^81^ strategy combines APEX-based PL with phosphorylation enrichment, enabling the identification of phosphorylation events near specific kinases under dynamic conditions. This approach has uncovered known and novel substrates of kinases such as MAPK1 and PKA. Similarly, the SubMAPP^82^ strategy integrates TurboID-based PL with photoactivation, allowing spatiotemporal control over subcellular phosphorylation mapping. By activating PL enzymes within specific organelles, SubMAPP has captured dynamic phosphorylation changes, extending its applications to primary neurons and live mice. Another study combined BioID-based PL with kinase perturbation and phosphorylation motif analysis to identify endogenous substrates of kinases such as CK2 and PKA^83^. This method enables direct identification of specific kinase substrates, addressing the limitations of indirect approaches.

Phosphoproteomics is an emerging field for interpreting complex regulatory mechanisms of biological systems. While traditional methods face limitations in spatial and temporal resolution, PL offers a powerful solution by enabling localized and time-resolved phosphoproteome profiling. By integrating PL techniques, future research can uncover novel regulatory networks and therapeutic targets.

An important consideration is whether PL interferes with the recovery of phosphopeptides. Because protein phosphorylation occurs primarily on serine, threonine and tyrosine residues, and biotin-based PL enzymes such as BioID and TurboID predominantly react with lysine residues, biotinylation is unlikely to substantially impact phosphopeptide recovery in these systems. By contrast, methods that generate phenol radicals—such as APEX or HRP—or those using photocatalytic labeling can potentially modify tyrosine residues. This may interfere with phosphotyrosine detection and affect overall phosphoproteome coverage. Therefore, the effects of residue-specific labeling chemistries on phosphoproteomic analysis warrants further investigation.

### Integration with imaging

To overcome the intrinsic limitations of spatial resolution in proteomic studies, researchers have integrated PL methods with imaging strategies. APEX systems improve spatial resolution by enabling high-contrast visualization through electron microscopy^29,31,84^. Peroxidases such as APEX and HRP exhibit efficient activity across various cellular compartments without the need for light. These systems provide precise labeling, facilitating ultrastructural studies and spatially resolved proteomic mapping, thereby addressing the limitations of conventional imaging techniques.

The newly developed FLEX system enhances PL by using low H_2_O_2_ concentrations to minimize oxidative stress while maintaining efficient phenol oxidation^85^. Unlike the conventional HRP system that requires large amounts of exogenous H_2_O_2_, FLEX uses the JFT1 fluorescent probe to generate stable, localized phenoxy radicals. This ensures robust labeling even during harsh processing, making it ideal for correlative light and electron microscopy and expanding its application to fixed sample analysis. By addressing the limitations of H_2_O_2_ supplementation and enhancing fluorescence signal retention, FLEX broadens the applicability of PL for mapping dynamic subcellular interactions in fixed samples.

Meanwhile, the essential role of endogenous H_2_O_2_ in mediating intercellular redox signaling has been suggested^86^. Building on this concept, HRP-based labeling approaches utilizing endogenously generated H_2_O_2_ have been developed, eliminating the need for external H_2_O_2_ treatment^87–89^. These approaches enable the identification of intercellular interaction networks without artificial perturbations. They can be utilized to capture and image intercellular interactions in processes such as neuronal signaling and remodeling.

Such advances have established PL as a valuable imaging tool complementing transcriptomic or proteomic data. This integration enables high-resolution mapping of protein interactions and modifications within intact tissues^90^. Ultimately, the integration of PL with imaging technologies further allows real-time visualization of molecular interactions at subcellular resolution, uncovering mechanisms of neural plasticity, connectivity and disease progression.

### Integration with multi-omics

The multi-omics approach is exemplified by the generation of the BRAIN Initiative Cell Census Network, which contains transcriptomic, epigenomic and spatial datasets that produce high-resolution atlases of neuronal and nonneuronal cell populations across the brain^91–93^. Among these approaches, spatial transcriptomics stands out as one of the essential analyses in neuroscience for understanding how various cell types contribute to specific brain functions^94^. However, transcriptomics remains limited to mRNA abundance patterns and fails to capture the protein interactions and posttranslational modifications critical for synaptic function^95–97^. Hence, integrating of transcriptomic and proteomic analyses is essential for a comprehensive understanding of neural networks^98,99^.

**Fig. 4:**
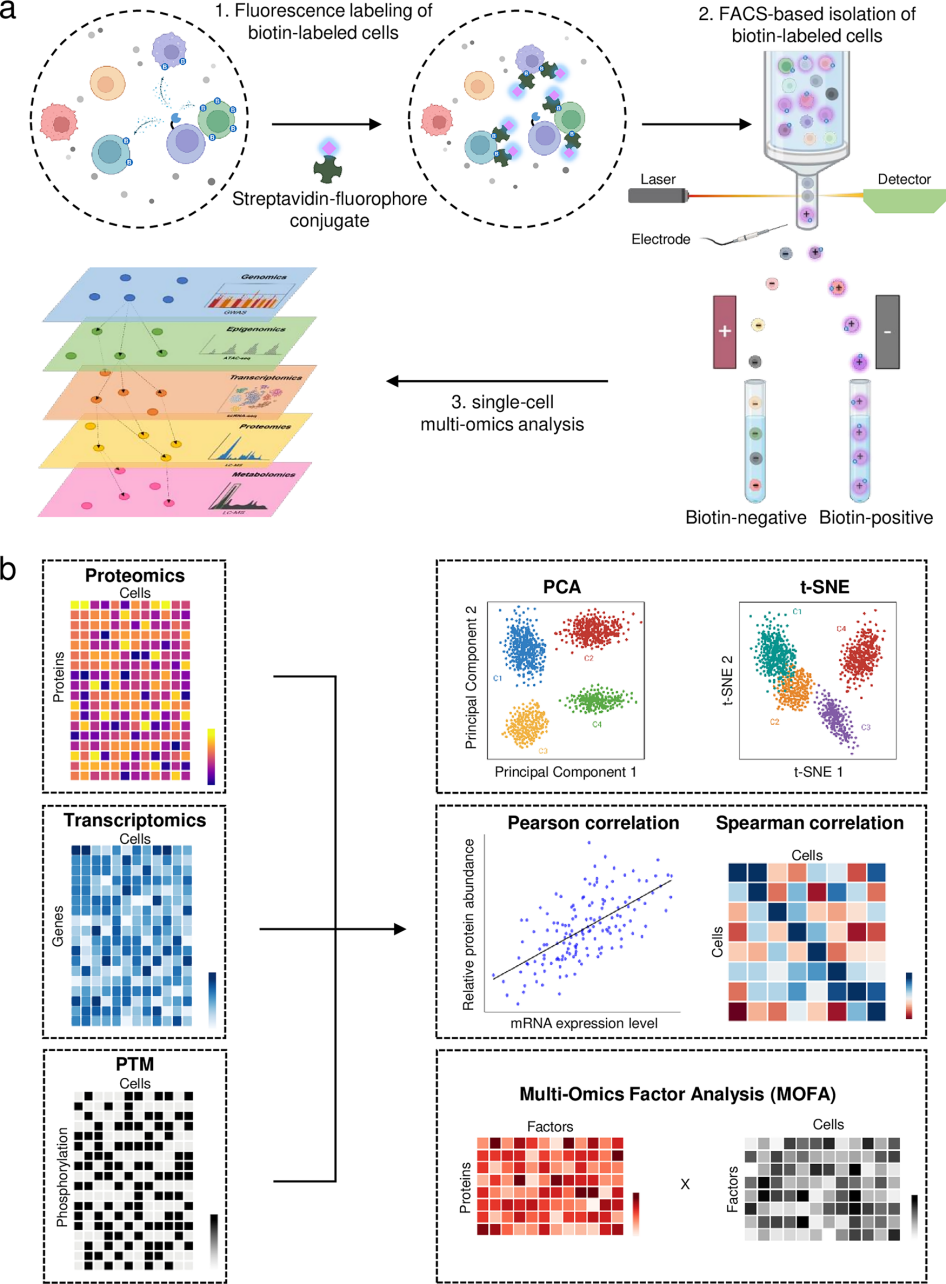
PL for multi-omics data integration and analysis. **a** PL is used to tag cells adjacent to a specific target cell, followed by fluorescence-activated cell sorting (FACS) to separate Biotin^+^ and Biotin^−^ cells. The labeled cells are identified on the basis of fluorescence signals, ensuring precise isolation of interacting populations. The sorted cells are then subjected to scRNA-seq and multi-omics analyses, including genomics, epigenomics, transcriptomics, proteomics and metabolomics. **b** PL enables the acquisition of proteomics and transcriptomics data, as well as phosphorylation-related information through phosphoproteomics, allowing the integration of these datasets to uncover latent factors not apparent at the surface. First, principal component analysis (PCA) and *t*-distributed stochastic neighbor embedding (t-SNE) are used to visualize the variability of each omics dataset and perform cell clustering. Second, Spearman and Pearson correlation analyses enable the investigation of gene–protein relationships, providing insights into the interplay between transcription and translation. Third, multi-omics data are transformed into matrices, and latent factors explaining sample differences are inferred using multi-omics factor analysis (MOFA), an unsupervised dimensionality reduction method. Created with BioRender.com.

PL can serve as a tool for multi-omics by connecting proteomics and transcriptomics. PL enables the isolation of the target cells by selectively labeling proximal cells, facilitating downstream multi-omics analyses such as scRNA-seq and single-cell proteomics (Fig. 4a). For example, in a study by Qiu et al.^15^, the FucoID system was used to label tumor-infiltrating lymphocytes and their interacting cancer cells. The labeled cells were sorted using fluorescence-activated cell sorting and analyzed through scRNA-seq, revealing molecular signatures involved in immune–tumor interactions. Similarly, Zhang et al.^16^ developed the QMID platform to record the spatial organization of immune cells within tissues with scRNA-seq. The PhoTag system facilitates multi-omics integration by using photoactivatable probes that selectively tag cells engaged in direct interactions^70^. These studies highlight PL’s role in multi-omics integration by mapping intercellular interactions while preserving spatial context.

In addition to connecting transcriptomics and proteomics, PL can also be extended to the epigenomic layer by profiling protein interactions at specific chromatin states or genomic loci. For instance, ChromID leverages engineered chromatin readers fused to biotin ligases to capture the local proteome associated with specific histone modifications in living cells^100^. Similarly, the GLoPro approach combines dCas9 with APEX2 to map proteins associated with defined genomic regions such as promoters, enabling targeted chromatin proteomics^101^. These studies demonstrate that PL not only preserves spatial context but also facilitates integration of epigenomic information. In addition, phenol radicals generated by peroxidases can be used to label and enrich specific metabolites, paving the way for the integration of metabolomic data in to spatially resolved molecular analyses.

To effectively integrate multi-omics single-cell data, various computational approaches have been developed to address the challenges of high-dimensionality, modality-specific noise and differences in data structure^102^ (Fig. 4b). Principal component analysis reduces dimensionality while preserving variance, identifying key variations across omics layers. More advanced techniques, such as *t*-distributed stochastic neighbor embedding and Uniform Manifold Approximation and Projection, enhance visualization by capturing structural relationships, aiding in cell clustering. Multi-omics factor analysis further integrates heterogeneous data by learning shared representations, revealing underlying biological relationships. These methods collectively align transcriptomic, proteomic and phosphoproteomic datasets, enabling a comprehensive understanding of coordinated molecular programs.

PL technologies bridge the gap between spatial biology and multi-omics analysis, offering a comprehensive view of cellular interactions in their native environments. By integrating multi-omics data obtained through these methods, researchers can construct detailed molecular maps that capture both intercellular communication and intracellular regulatory networks. This integrative approach holds substantial potential for neuroscience and may provide new insights into the molecular mechanisms underlying neurodevelopmental and neurodegenerative disorders.

## Conclusion

PL has advanced neuroscience research by enabling the precise identification of PPIs within living tissues. PL has unveiled previously hidden molecular networks that orchestrate synaptic development, plasticity and dysfunction. This has been achieved by integrating PL approaches with subcellular, cell-type-specific and cell-surface proteomic profiling. This ability to capture transient interactions in a spatial manner enhances our understanding of complex biological processes.

Recent methodological refinements have made PL more versatile, allowing researchers to target proteins in distinct neuronal compartments or label cell–cell contacts within intact brain tissue. Beyond charting local proteomes, PL has also shown promise in isolating cell subpopulations for transcriptomic profiling, enabling integrative multi-omics analyses. Such synergy can reveal the unique molecular signatures of neurons and glia and clarifies how diverse cell types coordinate during normal brain function or in pathological states.

Despite these advances, several technical challenges remain. Limited temporal resolution in some systems can hinder the detection of rapid interactions, while nonspecific background labeling and the need for rigorous data filtering complicate downstream analysis. These challenges highlight the importance of careful experimental design and data interpretation to ensure biological relevance and reproducibility. Addressing these issues requires optimized controls, balanced filtering strategies and site-resolved biotinylation analysis. Emerging photocatalytic and calcium-dependent PL strategies aim to address these issues by offering improved spatiotemporal control and reducing off-target effects. Furthermore, harnessing PL to drive phosphoproteomic investigations promises to uncover the dynamic phosphorylation events that govern rapid signaling changes in neurons.

As PL methods continue to mature, they hold immense potential to deepen our understanding of synaptic regulation, circuit assembly and disease mechanisms. Ultimately, these advancements could pave the way for precise, targeted interventions across a wide range of neurological disorders. Future PL applications will continue to contribute to bridging the gap between molecular and cellular neuroscience.
